# Emerging novel coronavirus (2019-nCoV)—current scenario, evolutionary perspective based on genome analysis and recent developments

**DOI:** 10.1080/01652176.2020.1727993

**Published:** 2020-02-27

**Authors:** Yashpal Singh Malik, Shubhankar Sircar, Sudipta Bhat, Khan Sharun, Kuldeep Dhama, Maryam Dadar, Ruchi Tiwari, Wanpen Chaicumpa

**Affiliations:** a Division of Biological Standardization, ICAR-Indian Veterinary Research Institute, Bareilly, Uttar Pradesh, India; b Division of Surgery, ICAR–Indian Veterinary Research Institute, Bareilly, Uttar Pradesh, India; c Division of Pathology, ICAR–Indian Veterinary Research Institute, Bareilly, Uttar Pradesh, India; d Razi Vaccine and Serum Research Institute, Agricultural Research, Education and Extension Organization (AREEO), Karaj, Iran; e Department of Veterinary Microbiology and Immunology, College of Veterinary Sciences, Deen Dayal Upadhayay Pashu Chikitsa Vigyan Vishwavidyalay Evum Go-Anusandhan Sansthan (DUVASU), Mathura, India; f Center of Research Excellence on Therapeutic Proteins and Antibody Engineering, Department of Parasitology, Faculty of Medicine Siriraj Hospital, Mahidol University, Bangkok, Thailand

**Keywords:** Coronavirus, 2019-nCoV, Severe Acute Respiratory Syndrome CoV, Middle East Respiratory Syndrome CoV, Public Health Emergency, genetic analyses, zoonoses, reservoir host, therapeutics, vaccines

## Abstract

Coronaviruses are the well-known cause of severe respiratory, enteric and systemic infections in a wide range of hosts including man, mammals, fish, and avian. The scientific interest on coronaviruses increased after the emergence of Severe Acute Respiratory Syndrome coronavirus (SARS-CoV) outbreaks in 2002-2003 followed by Middle East Respiratory Syndrome CoV (MERS-CoV). This decade’s first CoV, named 2019-nCoV, emerged from Wuhan, China, and declared as ‘Public Health Emergency of International Concern’ on January 30^th^, 2020 by the World Health Organization (WHO). As on February 4, 2020, 425 deaths reported in China only and one death outside China (Philippines). In a short span of time, the virus spread has been noted in 24 countries. The zoonotic transmission (animal-to-human) is suspected as the route of disease origin. The genetic analyses predict bats as the most probable source of 2019-nCoV though further investigations needed to confirm the origin of the novel virus. The ongoing nCoV outbreak highlights the hidden wild animal reservoir of the deadly viruses and possible threat of spillover zoonoses as well. The successful virus isolation attempts have made doors open for developing better diagnostics and effective vaccines helping in combating the spread of the virus to newer areas.

## 1. Introduction

**Figure 1.**
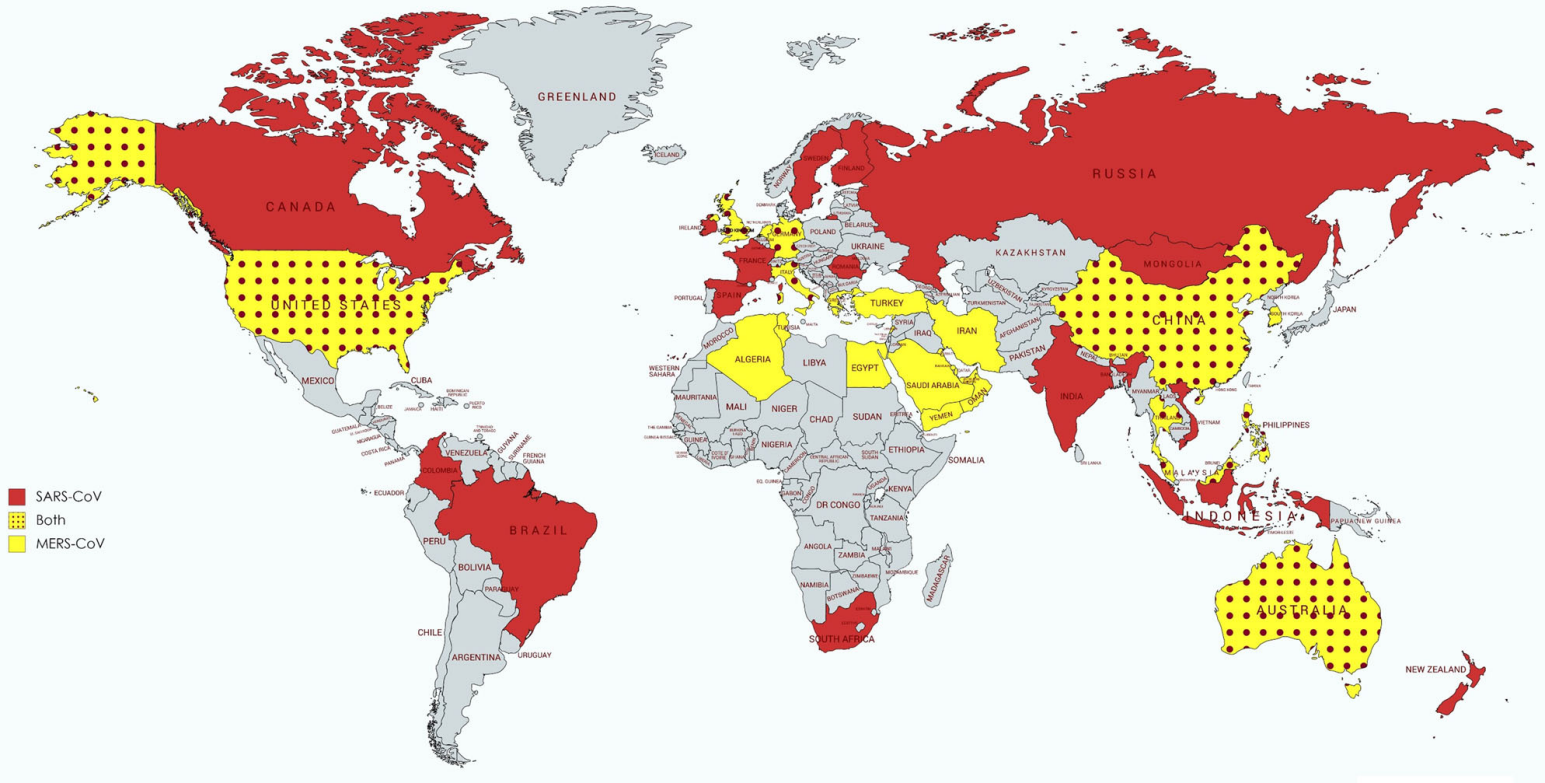
World-map depicting countries with SARS-CoV and MERS-CoV outbreaks. Red and yellow colors represent the global distribution of only MERS-CoV and SARS-CoV, respectively. The yellow-red dotted pattern shows the incidence of both viruses in the countries.

Coronaviruses (CoVs) are well-known causes of severe infections, respiratory, enteric and systemic, in humans and numerous animal hosts. The CoV infections have been reported in cattle, swine, horses, camels, rodents, cats, dogs, bats, palm civets, ferrets, mink, rabbits, snake, and several other wild animals and avian species (Fehr and Perlman 2015; Kahn and McIntosh 2005). The coronaviruses of relevant veterinary species are shown in Table 1 with organ affected and clinical signs. Though human CoVs were identified for the first time in the year 1960 from respiratory infections in adults as well as children, the major scientific interest in CoVs research grew only after the emergence of Severe Acute Respiratory Syndrome CoV (SARS-CoV) in the year 2002-2003 (Drosten et al. 2003; Ksiazek et al. 2003; Peiris et al. 2003). In this SARS-CoV epidemic, around 8000 confirmed human cases with 774 deaths (around 9.5% mortality rate) occurred that was a result of its global spread (Kahn and McIntosh 2005). Initially, the virus was detected in the caged Himalayan palm civets and these were thought to be the natural host of this virus (Guan et al. 2003). Following SARS-CoV incidence in 2003, a similar CoV named HKU3-1 to HKU3-3 were identified in the horseshoe bats (non-caged) in 2005 from Hong Kong (Lau et al. 2005). Since then, bats are considered to be the natural host and potential reservoir species that could be held responsible for any future CoVs epidemics and/or pandemics (Cui et al. 2019, Li et al. 2005). After the 2003 and 2005 SARS-CoV epidemics, an analogous virus emerged in the Middle East region of the world leading to severe respiratory illness and was named the Middle East Respiratory Syndrome CoV (MERS-CoV) (Zaki et al. 2012). The mortality was higher than previous SARS-CoV pandemic claiming around 919 lives out of the total 2521 human cases (around 35% mortality) (World Health Organization 2015). Notably, dromedary camels were connected with the transmission of MERS-CoV (Alagaili et al. 2014). Further, its origin was also traced from bats (Ithete et al. 2013). All these highly pathogenic human CoVs, SARS and MERS, show emergence over wider areas of the world posing high risk of human-to-human transmission and fatal consequences thereto (Figure 1).

**Table 1.** Coronaviruses of relevant veterinary species.

Host	Virus	Genus	Organ affected	Clinical sign
Bovine	Bovine coronavirus	*Betacoronavirus*	GI Tract Respiratory tract	Calf diarrhea Winter dysentery (bloody diarrhea) in adult cattle Bovine respiratory disease complex (shipping fever)
Porcine	Transmissible gastroenteritis (TGE) virus	*Alphacoronavirus*	GI Tract	Profuse watery yellow diarrhea, Vomition, dehydration
	Porcine respiratory coronavirus	*Alphacoronavirus*	Respiratory tract	Mild respiratory disease or subclinical
	Porcine epidemic diarrhea virus (PEDv)	*Alphacoronavirus*	GI Tract	Watery diarrhea, vomition, dehydration
	Porcine hemagglutinating encephalomyelitis virus	*Betacoronavirus*	GI Tract CNS	Vomition, Diarrhea Wasting disease Encephalomyelitis
	Porcine deltacoronavirus	*Deltacoronavirus*	GI Tract	Gastroenteritis
Feline	Feline enteric coronavirus	*Alphacoronavirus*	GI Tract	Mild gastroenteritis and diarrhea
	Feline infectious peritonitis virus	*Alphacoronavirus*	Respiratory tract Abdominal cavity CNS	Pneumonia Peritonitis Neurological disorder
Canine	Canine coronavirus	*Alphacoronavirus*	GI Tract	Mild to severe gastroenteritis
	Canine respiratory coronavirus	*Betacoronavirus*	Respiratory tract	Kennel cough Pneumonia
Equine	Equine coronavirus	*Betacoronavirus*	GI Tract	Gastroenteritis
Camel	Middle East respiratory syndrome (MERS) coronavirus	*Betacoronavirus*	Respiratory tract	Mild respiratory signs
Chicken	Avian infectious bronchitis virus	*Gammacoronavirus*	Trachea Kidney Reproductive tract	Tracheobronchitis, Rales Nephritis Decreased egg production

This decade’s first CoV emergency was from Hubei province of China, and as on February 4, 2020, 425 deaths have been reported in China only (World Health Organization 2020b). Further, the spread of this novel coronavirus, named 2019-nCoV, has been noted in 24 countries till date. Considering the global threat of the 2019-nCoV, the World Health Organization (WHO) declared it as a ‘Public Health Emergency of International Concern’ on January 30^th^, 2020.

This rapid communication provides an overview of the recently emerging coronavirus (2019-nCoV) with regards to its current scenario, comparative analysis with respects to previously reported CoVs, evolutionary perspective based on genome analysis while covering the recent advances on vaccines and therapeutics in brief.

## 2. Coronaviruses

Coronaviruses (CoVs) constitute a large family of viruses found in nature. CoVs belongs to the family *Coronaviridae* and the order *Nidovirales* possessing a single-stranded, positive-sense RNA genome ranging from 26 to 32 kb in length (the largest genome of known RNA viruses) with G + C contents varying from 32 to 43%. Based on the genomic structure and phylogenetic analysis the subfamily *Orthocoronavirinae* consists of four genera namely *Alphacoronavirus*, *Betacoronavirus*, *Gammacoronavirus* and *Deltacoronavirus*. Among these *Alphacoronavirus* and *Betacoronavirus* infects only mammals and are responsible for respiratory infection in humans and enteritis in animals. Two major zoonotic pathogenic coronaviruses, SARS-CoV and MERS-CoV belong to the genus *Betacoronavirus*. The genus *Betacoronavirus* further distributed in five subgenus among which *Sarbecovirus* contains SARS-CoV and the novel coronavirus (2019-nCoV) (de Groot et al. 2012). The other subgenera under *Betacoronavirus* are *Embecovirus, Hibecovirus, Merbecovirus*, and *Nobecovirus*.

The nCoV contains at least six open reading frames (ORFs) and many other accessory genes like other CoVs. The 5′ terminal two-thirds of the genome contains two open reading frames (ORFs), ORF1 and ORF2 which encodes two polyproteins, pp1a and pp1ab, which is further cleaved into 11 and 16 proteins, respectively. These 16 mature proteins are responsible for several important functions in genome maintenance and virus replication. The structural proteins namely spike (S), an envelope protein (E), membrane protein (M) and nucleocapsid (N) are located at the one-third 3′ terminal of the genome. In addition to these genes, there are several accessory proteins which help in virus replication. The S gene is one of the most important genes for receptor binding and host specificity. Some coronaviruses have hemagglutinin-esterase (HE) protein in their virion.

## 3. The emergence of novel-Coronavirus (2019-nCoV)

**Figure 2.**
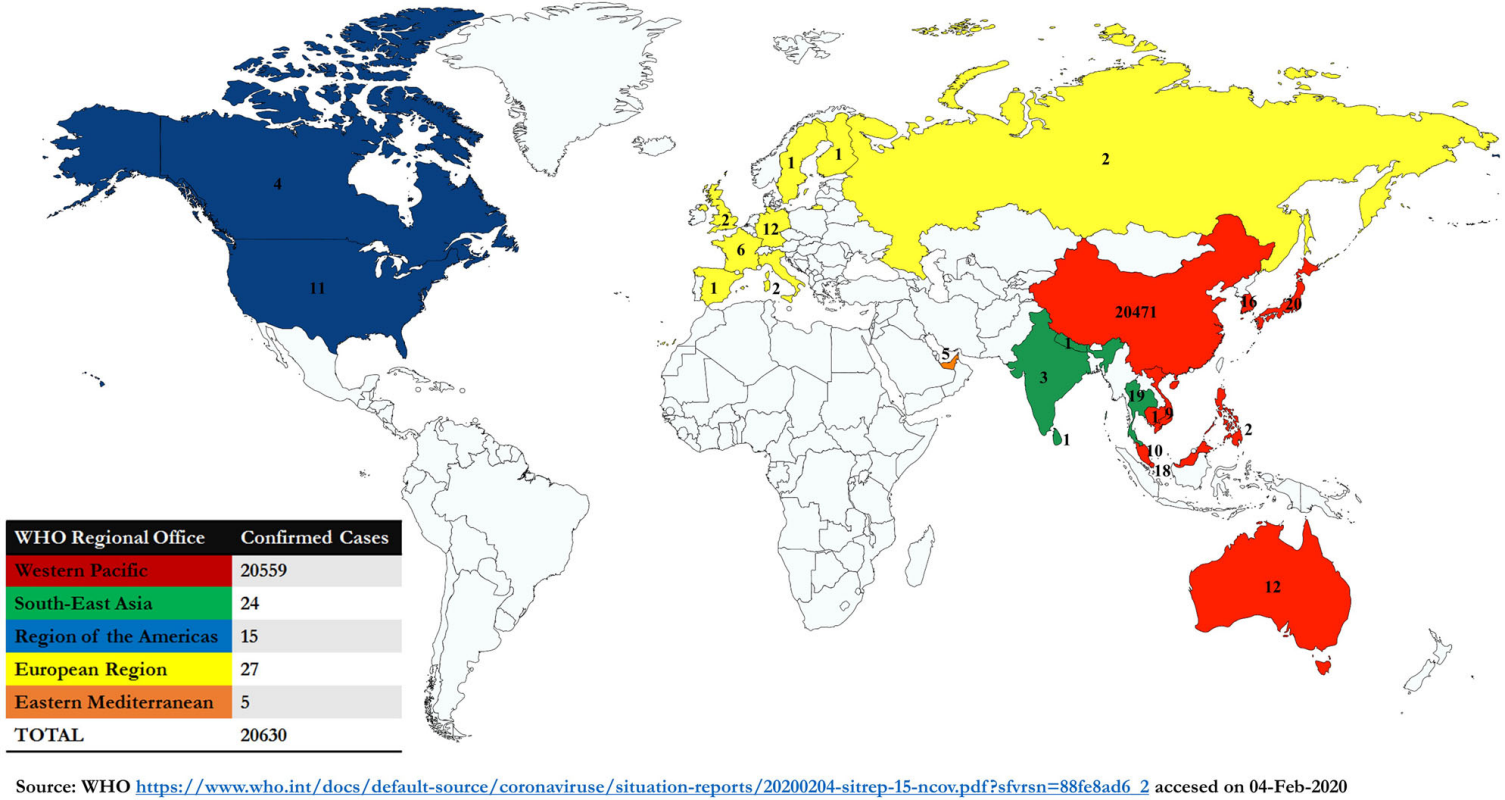
Countries, territories or regions with reported confirmed cases of 2019-nCoV, February 4^th^, 2020. Different colors indicate different geographical regions with the number of confirmed cases. In the table, region-wise total number of confirmed cases are depicted.

During the first week of December 2019, a few cases of pneumonia appeared in the city of Wuhan, Hubei province of China. The patients exhibited a history of visiting the local nearby Huanan seafood market which deals in the sale of different live animals, where zoonotic (animal-to-human) transmission suspected as the main route of disease origin (Hui et al. 2020). Firstly, the affected patients presented with pneumonia-like symptoms, followed by a severe acute respiratory infection. Some cases showed rapid development of acute respiratory distress syndrome (ARDS) followed by serious complications in the respiratory tract. On Jan 7^th^, 2020, it was confirmed by the Chinese Center for Disease Control and Prevention (CDC) that a new coronavirus has emerged and was named 2019-nCoV. As on February 4^th^ 2020, China has confirmed 20471 cases with 425 deaths and 2788 severe cases of 2019-nCoV. In addition to China, 24 different countries from Europe, Northern America, Southeast Asia, Eastern Mediterranean, and Western Pacific Asia have reported the confirmed cases of this disease making the total tally of confirmed cases to 20630 worldwide (Figure 2). Although the mortality rate due to 2019-nCoV is comparatively lesser than the earlier outbreaks of SARS and MERS-CoVs, as well as this virus presents relatively mild manifestations, the total number of cases are increasing speedily and are crossing the old census. There is a high risk of human-to-human transmission which has also been reported in family clusters and medical workers. The infected patients with nCoV exhibit high fever and dyspnea with chest radiographs showing acute invasive lesions in both lungs.

## 4. Phylogenetic analysis and sequence identity

Currently, knowledge about the origin of the virus, its particular receptor, clinical spectrum and host species preference is unknown. World over scientists are engaged in unraveling the genomic nature of this virus. As of now, around 15 complete 2019-nCoV genome sequences (10 China and 5 USA) have been reported. To note, a report based on the codon usage analysis speculated snakes to be its natural host, whereas several subsequent reports after that publication rejected this hypothesis. They found the nearest members to be Bat_SARS-like CoVs (Bat-SL-CoVs, MG772933 & MG772934) in phylogenetic analysis within the subgenus S*arbecovirus*.

### 4.1. Analysis targeting the complete genome of 2019-nCoVs

**Figure 3.**
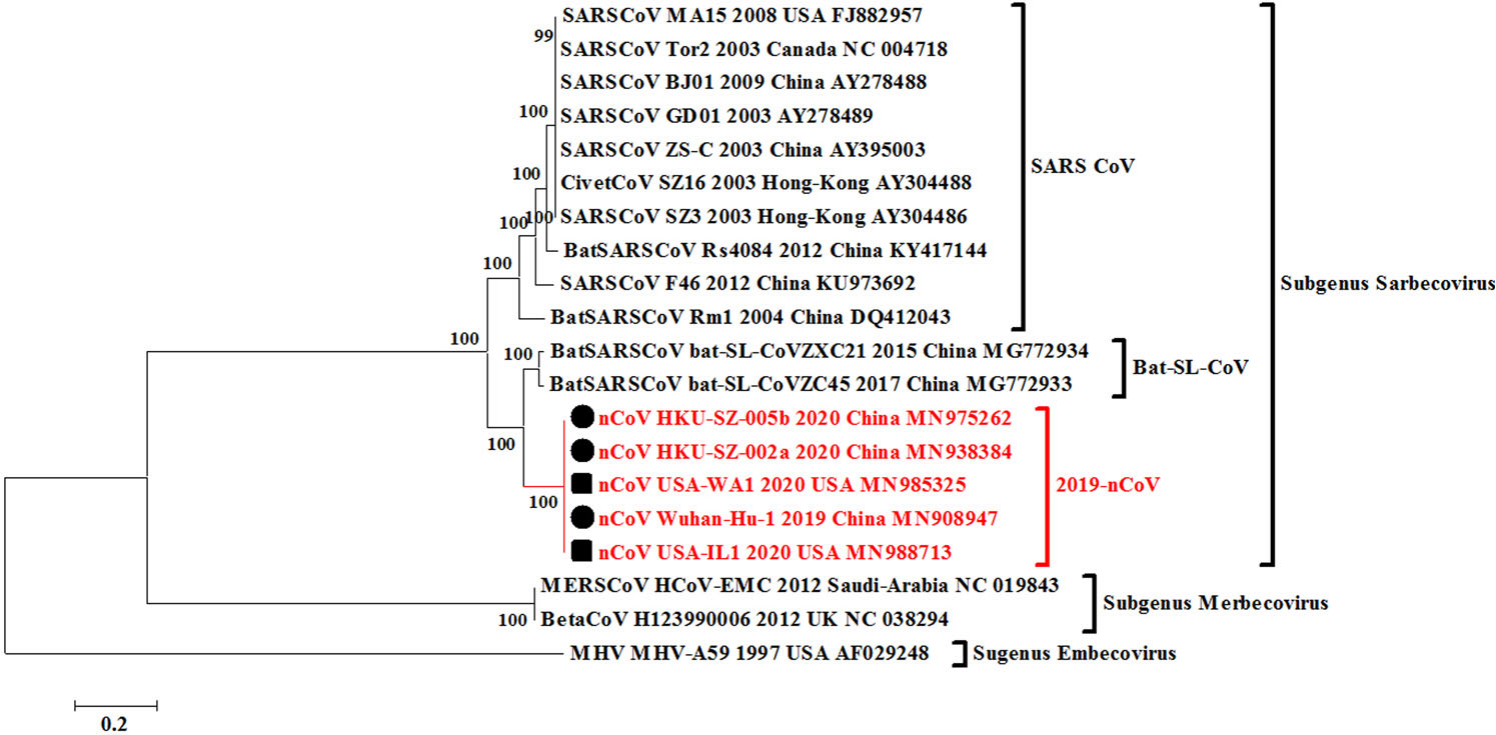
Phylogenetic analysis of 2019-nCoV isolates using complete genomes. The 2019-nCoV isolates analyzed with related CoVs from past human outbreaks and of animal origin. The solid-black circles are for nCoV isolates from China and solid-black squares are for the isolates from the USA.

In this study, we also attempted to reveal the evolutionary perspective of the recently emerging 2019-nCoV based on the complete genome analysis. Phylogenetic analysis was done using the MEGA 7.0 version applying the Maximum likelihood method (ML) based General Time Reversible substitution model with the available whole genome sequences of 2019-nCoV available in the NCBI GenBank database till January 28^th^, 2020. Pairwise identity of the current 2019-nCoV outbreak sequences was calculated using the MegAlign software of DNASTAR. In the whole genome phylogenetic analysis, the 2019-nCoV strains from China and the USA clustered in a monophyletic clade (Figure 3). The nearest neighbors of the 2019-nCoV isolates from China and USA were two Bat_SARS-like coronaviruses (Bat-SL-CoVZC45, Accession no. MG772933 and Bat-SL-CoVZXC21, Accession no. MG772934). These two Bat_SARS-like CoVs shared a 100% bootstrap support with 2019-nCoV strains of the current outbreak. Using the MegAlign and MEGA 7.0 software based Clustal W alignments, the nucleotide sequence identity of 2019-nCoV strains revealed the highest similarity of greater than 88.2% with two Bat_SARS_like CoVs. These findings were in accordance with report of Zhu and colleagues where a nearby sequence identity of 86.9% with previously published Bat_SARS-like CoV was reported (Zhu et al. 2020). Contrarily, the genome of 2019-nCoV has also been reported to be 96% identical to the bat coronavirus based on Simplot analysis where it has been found closer to bat CoV isolate RaTG13 previously detected in *Rhinolophus affinis* (intermediate horseshoe bat) from Yunnan Province, indicating its origin from the bats (Zhou et al. 2020). Based on the available information it is rather early to predict the origin of this novel coronavirus without a comprehensive analysis of emerging nCoV strains from different parts of the world. To note, the sequence identity based on the complete genome sequences between current outbreaks 2019-nCoV isolates from China and the USA ranges 99.8 to 100% on the nucleotide level indicating their common origin of evolution.

### 4.2. Analysis targeting the spike (S)-gene of CoVs

**Figure 4.**
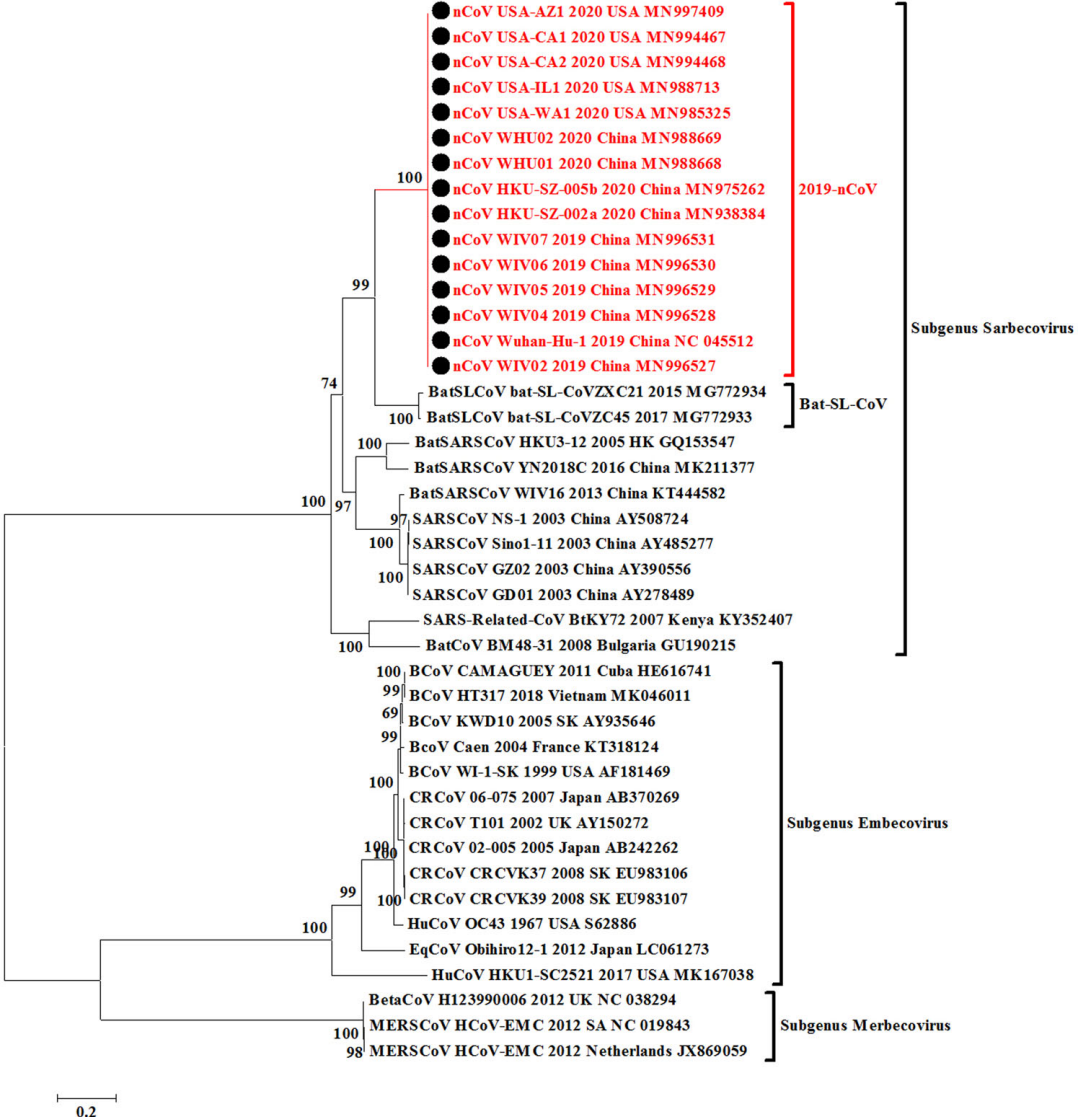
Spike (S) glycoprotein gene-based phylogenetic analysis of 2019-nCoV isolates (10 Chinese and 5 USA isolates). The 2019-nCoV isolates analyzed with related CoVs from past human outbreaks and of animal-origin including MERSV (camel), bovine coronavirus, canine coronavirus, bat_coronaviruses. The solid-black circles are for nCoV isolates from China and the USA.

We furthermore extended the analysis targeting the Spike (S) glycoprotein gene of the CoVs from human SARS, animal-origin CoVs including MERSV (camel), bovine coronavirus, canine coronavirus, bat_coronaviruses and the current outbreak nCoVs from different regions. The sequences of nCoV available in the NCBI GenBank database till January 28^th^, 2020 were retrieved. Phylogenetic analysis was done using the MEGA 7.0 version applying the Maximum likelihood method (ML) based General Time Reversible substitution model with gamma distribution. Pairwise identity of the current 2019-nCoV outbreak sequences was calculated using the MegAlign software of DNASTAR. In the S-gene based phylogeny, 10 Chinese and 5 USA nCoV isolates (Figure 4) revealed that all the isolates are nearly identical across the S-gene based phylogeny constituting a monophyletic clade (Figure 4). The two Bat_SARS-like CoVs (Bat-SL-CoVZC45, MG772933 and bat-SL-CoVZXC21, MG772934) shared 100% bootstrap support with 2019-nCoV isolates of the current outbreaks. Based on the MegAlign and MEGA 7.0 software based Clustal W alignments, the sequence identity of 2019-nCoV strains revealed Bat SARS-like CoVs (Bat-SL-CoVZC45, MG772933 and bat-SL-CoVZXC21, MG772934) as the nearest neighbors with 77.6 to 78.2% sequence identity on nucleotide basis. In the phylogenetic tree, the current outbreak nCoV isolates were fairly distinct than the previously reported SARS-CoV or BatSARS-CoV strains but were clustering inside a common major clade which includes strains from subgenus *Sarbecovirus.* The per cent identity with other SARS-CoV or BatSARS-CoV strains was 70.8 to 74.7% (Supplementary data 1). The per cent similarity on nucleotide basis between nCoV isolates and canine respiratory coronaviruses (CRCoV) and bovine coronaviruses (BCoV) of subgenus *Embecovirus* ranged between 40.8 to 41.5%. Furthermore, the per cent identity of nCoV isolates was found lower (40.2%) with the mild respiratory human coronavirus isolate HCoV-OC43 of same subgenus *Embecovirus* containing animal-origin coronaviruses.

Additionally, for S-gene, per cent similarity range pattern was evaluated in amino acid based index where 2019-nCoV isolates from China and USA were 100% identical. The range varied between 81.2 to 81.8% and 77.0 to 78.1% for 2019-nCoV isolates with Bat_SARS-like CoVs and other SARS like CoVs, respectively. The per cent identity with human coronavirus isolate HCoV-OC43 was also found lower (28.0%) (Supplementary data 2).

Overall, the nucleotide and amino acid based per cent identities indicate toward highly diverged nature of novel coronaviruses. To note, in the phylogenetic tree the 2019-nCoV isolates classified in subgenus *Sarbecovirus* and the sequences from CRCoV, BCoV and HCoV-OC43 isolates clustered in subgenus *Embecovirus*.

**Figure 5.**
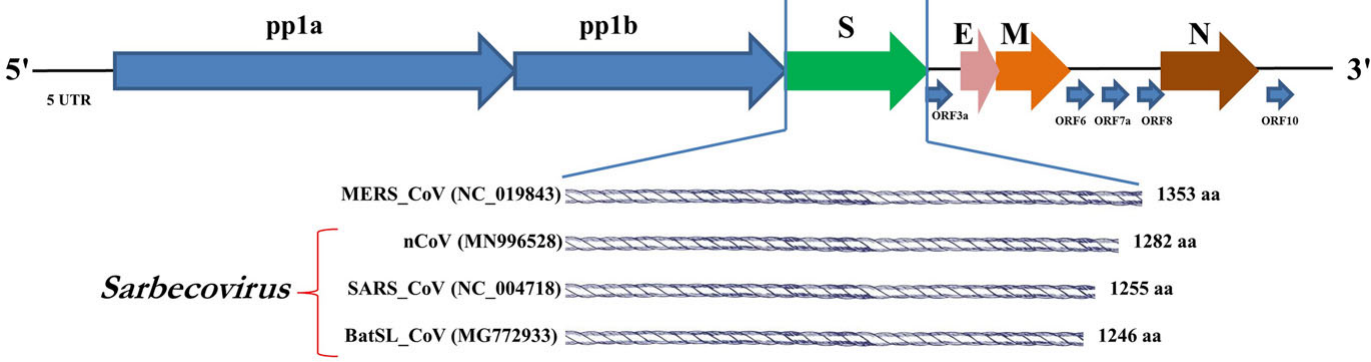
Genome organization of novel coronavirus. Comparative spike (S) protein sequence length of different *Betacoronaviruses* is depicted.

In the genomic analysis, the S gene of 2019-nCoVs was found to exhibit lower sequence identity with other *Betacoronaviruses*. Difference in spike protein encoded by 2019-nCoV compared with the Bat SARS-like CoVs, SARS-CoV, and MERS-CoV is depicted in Figure 5. The length of ‘S’ protein of nCoV is longer (1282 amino acids) than other two viruses (SARS-1255 amino acid and BatSL-1246 amino acids) under the same *Sarbecovirus* subgenus. The ‘S’ protein of nCoV has been detected with three short insertions at the N-terminal region, along with four changes in the receptor binding motif inside the receptor binding domain in comparison with SARS-CoV (Zhou et al. 2020). Several previous studies have shown the usage of different receptors for CoVs like human ACE2 for SARS-CoV and CD26 for MERS-CoV (Lu et al. 2020). Identical motifs and residues to that of SARS-CoVs have been identified in the nCoV, yet they show divergence based on phylogeny (Letko and Munster 2020). A recent finding proves that the binding capacity of nCoV ‘S’ protein with human ACE2 is as efficient as SARS-CoV, which further promotes the human-to-human transmission (Letko and Munster 2020).

## 5. Advances and prospects in developing vaccines and therapeutics

Several attempts have been made to develop vaccines against human coronavirus infection in the past decades. But the degree of cross-protection provided by such vaccines is greatly limited due to the extensive diversity in antigenic variants even within the strains of a phylogenetic sub-cluster (Graham et al. 2013). As for MERS and SARS coronaviruses, there is no licensed specific antiviral treatment or vaccine available till now. However, few of the advances made in developing vaccines and therapeutics for SARS-CoV and MERS-CoV could be exploited for the countering 2019-nCoV. But since the efforts to design and develop any vaccine or antiviral agent to tackle the presently emerging coronavirus pathogen would take some time, therefore till then we need to rely extensively on enforcing highly effective prevention and control measures to minimize the risk of 2019-nCoV transmission and spread to the best feasible extent (Cheng et al. 2020). Majority of the vaccines that are being developed for coronaviruses targets the Spike glycoprotein or S protein (Graham et al. 2013). This is mainly because of the fact that S protein is the major inducer of neutralizing antibodies (Jiang et al. 2005). Several kinds of vaccines and antiviral drugs that are based on S protein have been previously evaluated. Among them, the S protein-based vaccines include full-length S protein vaccines, viral vector-based vaccine, DNA-based vaccine, recombinant S protein-based and recombinant RBD protein-based vaccines. Whereas S protein based antiviral therapies include RBD–ACE2 blockers, S cleavage inhibitors, fusion core blockers, neutralizing antibodies, protease inhibitors, S protein inhibitors, and small interfering RNAs (Du et al. 2009). Even though such therapeutic options have proven efficacy in the *in vitro* studies, however most of these haven’t undergone randomized animal or human trials and hence are of limited use in our present 2019-nCoV scenario. Remdesivir is a novel nucleotide analog prodrug that was intended to be used for the treatment of Ebola virus disease. It also has anti-coronavirus activity due to its inhibitory action on the SARS-CoV and MERS-CoV replication (Sheahan et al. 2017). At present, efforts are being made to identify and develop monoclonal antibodies that are specific and effective against 2019-nCoV. Combination therapy with 2019-nCoV specific monoclonal antibodies and remdesivir can be considered as the ideal therapeutic option for 2019-nCoV (Cohen 2020). Further evaluation is required before confirming the efficacy of such combination therapy. A variety of different therapeutic and vaccine designing approaches against coronaviruses are being explored and yet to be evaluated in terms of their potency, efficacy and safety, but hopefully the process of evaluation will be accelerated in the coming days (Cyranoski 2020; Lu 2020; Pillaiyar et al. 2020; Zaher et al. 2020).

## 6. Prevention and control measures

Seeing the possible role of animals in 2019-nCoV infection, WHO in its advice for public recommended to avoid the unprotected contact with both farm and wild animals (World Health Organization 2020a). The live-animal markets such as in China could provide chances to animal CoVs to get transmitted to humans and these markets may act as critical places for the origin of novel zoonotic pathogens and pose high public health risks during an outbreak.

The emergency pathogens could be counteracted by opting immediate and timely international collaborative efforts, cooperative efforts between human and animal health sectors. Other effective measures include One health approach, implementation of effective prevention and control strategies, rapid communication and networking, and exploring advances in science and technology for developing rapid and confirmatory diagnostics, enhancing disease surveillance and monitoring, implementation of strict biosecurity measures, and timely efforts toward designing appropriate and effective vaccines and therapeutics (Cheng et al. 2020; Cohen 2020; Cyranoski 2020; Lu 2020; Munjal et al. 2017; Singh et al. 2017).

In the present scenario of not having any direct acting anti-viral agent and vaccines, strict implementation of high vigilance for 2019-nCoV and appropriate prevention and control measures are of utmost importance to check the further spread and control of this virus (Cheng et al. 2020). Researchers and Authorities (WHO, CDC Atlanta and others) across the globe are working to combat the current ongoing 2019-nCoV outbreaks, identifying the possible origin of this novel virus, and to design and develop effective vaccines and therapeutics (Cohen 2020, Cyranoski 2020, Lu 2020, Mahase 2020). Studying the virus in details, its molecular biology and immunology, adaptive genetic changes, mutations and recombination events, elucidating clinical pathology and pathogenesis, identifying the route of origin, role of any mixing vessels (like birds, pigs, and mammals), jumping the species barrier, zoonotic potential, human-to-human transmission events, altogether would pave ways for designing effective prevention and control measures to counter 2019-nCoV

## 7. Conclusion and future prospects

The nCoV is the most recently emerging virus after the past episodes and panics haunted by Ebola, Zika and Nipah viruses, as well as earlier emergencies posed by Bird flu and Swine flu viruses. The emerging novel coronavirus (2019-nCoV) has become a global concern within a short span of time. Since its origin from Wuhan, Hubei province of China in the first week of December 2019, it has claimed 425 lives in China and infected 20630 in 24 countries including China. Additionally, new cases are emerging in different countries and three confirmed cases from India also emerged till compilation of this paper, February 4th, 2020. As of now deaths were reported only from China but now causality in Philippines reported for the first time outside China. The nCoV crisis has been declared as ‘Public Health Emergency of International Concern’ by WHO. The zoonotic route (animal-to-human) is suspected as the route of disease origin. Bats are considered as the natural reservoir hosts and play a crucial role in transmitting various viruses, including Ebola, Nipah, Coronavirus and others (Cui et al. 2019). A high diversity among zoonotic *Alphacoronaviruses* and SARS-CoV-related *Betacoronaviruses* has been found in the circulating bats of Western Europe (Gouilh et al. 2018). The genetic analyses predict bats as the most probable origin of 2019-nCoV. The diversity of CoVs in the bat population needs further investigation in details, as well as the surveillance and monitoring of bats becomes critical to prevent future outbreaks in animals and the public. The recent nCoV outbreak highlights the hidden wild animal reservoir of the deadly viruses and possible threat of spillover zoonoses. Successful virus isolation attempts have made doors open for developing better diagnostics and effective vaccines. A report emerged from China where the scientists claimed successful isolation of the 2019-nCoV virus in Vero and Huh7 cell lines from infected patients. Subsequently, in aiding the further research for designing rapid diagnostics and vaccine development for nCoV, scientists at The Peter Doherty Institute for Infection and Immunity at Melbourne, Australia, were also successful in growing the Wuhan coronavirus in cell culture. Further research is warranted on establishing animal models for the current 2019-nCoV unrevealing viral events of replication, transmission, and pathogenesis in humans. This could provide clues for discovering effective therapeutic regimens and vaccine testing purposes

## Supplementary Material

Supplemental Material
